# Synthesis, Characterization, and Supercapacitor Performance of a Mixed-Phase Mn-Doped MoS_2_ Nanoflower

**DOI:** 10.3390/nano12030490

**Published:** 2022-01-29

**Authors:** Ismaila T. Bello, Kabir O. Otun, Gayi Nyongombe, Oluwaseun Adedokun, Guy L. Kabongo, Mokhotjwa S. Dhlamini

**Affiliations:** 1 Department of Physics, College of Science, Engineering and Technology, University of South Africa, Johannesburg 1710, South Africa; gayinyongombe@gmail.com (G.N.); geekale@gmail.com (G.L.K.); 2 Department of Pure and Applied Physics, Ladoke Akintola University of Technology, Ogbomoso 4000, Nigeria; oadedokun@lautech.edu.ng; 3 Institute for the Development of Energy for African Sustainability, College of Science, Engineering and Technology, University of South Africa, Johannesburg 1710, South Africa; otunkabir2705@gmail.com; 4 Department of Chemistry and Industrial Chemistry, Kwara State University, Malete 241103, Nigeria

**Keywords:** supercapacitors, Mn-doped MoS_2_ nanoflowers, electrode materials, specific capacitance, energy density, power density

## Abstract

The fascinating features of 2D nanomaterials for various applications have prompted increasing research into single and few-layer metal dichalcogenides nanosheets using improved nanofabrication and characterization techniques. MoS_2_ has recently been intensively examined among layered metal dichalcogenides and other diverse transition metal-based materials, that have previously been studied in various applications. In this research, we report mixed-phase Mn-doped MoS_2_ nanoflowers for supercapacitor performance studies. The confirmation of the successfully prepared Mn-doped MoS_2_ nanoflowers was characterized by XRD, SEM-EDS, RAMAN, and BET research techniques. The mixed-phase of the as-synthesized electrode material was confirmed by the structural changes observed in the XRD and RAMAN studies. The surface area from the BET measurement was calculated to be 46.0628 m^2^/g, and the adsorption average pore size of the electrode material was 11.26607 nm. The electrochemical performance of the Mn-doped MoS_2_ electrode material showed a pseudo-capacitive behavior, with a specific capacitance of 70.37 Fg^−1^, and with a corresponding energy density of 3.14 Whkg^−1^ and a power density of 4346.35 Wkg^−1^. The performance of this metal-doped MoS_2_-based supercapacitor device can be attributed to its mixed phase, which requires further optimization in future works.

## 1. Introduction

Electrochemical energy storage devices are currently attracting considerable consideration for harnessing their energy potential, across the scientific world. This is due to the ever-increasing demand for eco-friendly energy storage devices (fuel cells, batteries, and supercapacitors), as a better replacement for the existing energy sources (fossil fuel) that are deteriorating climatic conditions around the world [1,2,3]. The seasonal recurrence of conventional forms of renewable energy resources, such as solar, hydel, wind, biomass, and tidal have made the storing mechanism of such energies vital. Therefore, the fast depletion of these energy resources based on their availability calls for the development of alternative energy storage devices [4]. The desire to produce a storage device that has a fast charge–discharge ability, with stable cyclic performance, safe operation, and cost-effectiveness led to the development of supercapacitors. Supercapacitors are high-power energy storage devices that possess a better capacitance output than conventional capacitors [5]. In the field of energy harvesting devices, the supercapacitor is one of the most researched topics. High energy/power density, fast charge/discharge capability, and excellent cycling stability are some of the distinguishing characteristics of supercapacitors that make them an appealing candidate technology [5,6,7,8,9]. Based on their storage mechanisms, supercapacitors are classified into electrochemical double-layer capacitors (EDLC) and pseudo-capacitor. An EDLC stores charge via the accumulation of electrolyte ions at the interface of the electrode–electrolyte. At the electrode–electrolyte interface, no charge is transferred, which can store charges by non-faradaic reactions. The second category is the pseudo-capacitors, which use the redox reaction system to store charge between the electrode and electrolyte. The faradaic reactions are used to transfer charges for energy storage applications [10,11,12].

Recently, carbon-based materials such as activated carbon, carbon black, graphene, carbon nanotubes, and their derivatives have been commonly used as active materials in designing EDLC devices, because of their good conductivity and excellent stability [4,11]. However, graphene’s strong hydrophobicity, poor dispersity, and aggregation properties prevent it from being used as an efficient supercapacitor electrode material [13,14,15]. Various transition metal sulfides, such as copper sulfide, nickel sulfide, zinc sulfide, molybdenum sulfide, manganese sulfide, strontium sulfide, and vanadium sulfide have attracted attention for supercapacitor applications, due to their natural abundance, easily controlled morphologies, multiple valences, and appropriate bandgap widths. Among these, MoS_2_ is a potential electrode material for supercapacitor applications, due to its intriguing sheet-like structure, which offers a large surface area for double-layered charge storage and higher intrinsic fast ionic conductivity [4,16,17,18]. The analogous structure of molybdenum sulfide (MoS_2_) with that of graphene means it has been considered for various energy storage, optoelectronics, sensing, and photocatalysis applications, owing to their unique morphology, excellent mechanical, and electrical properties [12,19,20,21,22]. MoS_2_ comprises covalently bonded S-Mo-S atoms, which are held together by weak van der Waals forces, with a higher capacity (theoretical) than graphite [23,24]. It has different polytype structures of 2H and 1T phases, coupled with variable oxidation states of +2 and +6. It also possesses a higher in-plane ionic and electrical conductivity than oxide [25,26]. Through the faradaic charge transfer mechanism, MoS_2_ can store charge in both an inter- and intra-layer. As a result, MoS_2_ and its composite are viable materials for high-performance pseudo-capacitor electrodes [9,12]. Considerable attention has been paid to MoS_2_, to improve its charge storage performance and to circumvent the interlayer self-aggregation because of the S-M-S interlayer van der Waals force. Therefore, the adjustment of its structural design and microscopic morphology and its composite optimization is needed to improve the super-capacitive behavior. Several findings have been reported on successfully prepared composite materials with activated carbon [27,28], metal oxides [29,30], graphene [31,32,33], metal hydroxide [34,35], conducting polymers [36,37,38], carbon nanotube [8], and metal sulfides [39,40]. However, there are few reported works on metal-doped composites of MoS_2_-based supercapacitors. Nickel-doped MoS_2_ nanosheets were reported by Palanisamy and colleagues for the fabrication of an asymmetric supercapacitor with the specific capacitance of 286 Fg^−1^ at 1 Ag^−1^, using three electrode systems in 1 M Na_2_SO_4_ [41]. Falola et al., 2017, reported a specific capacitance of 502 Fg^−1^ at 1Ag^−1^, using a three-electrode configuration in 1 M Na_2_SO_4_, from the copper doped MoS_2_ film [42]. Two system electrode configurations were also employed to study the electrochemical performance of platinum doped MoS_2_ nanosheets, as reported by Shao and co-workers [43]. The specific capacitance of 200.87 Fg^−1^ at 1 Ag^−1^ in a 1 M concentration of Na_2_SO_4_ electrolyte was obtained. Recently, Singha et al., 2020, obtained a specific capacitance of 88 Fg^−1^ at 1 Ag^−1^ in 2 M KOH, using a two-electrode configuration system, with Manganese doped MoS_2_ nanoflower as an active material [44]. More recently, cobalt doped MoS_2_ nanosheets and nanoflowers were, respectively, reported by Sun et al., 2018, and Rohit et al., 2021. The specific capacitance of 510 Fg^−1^ at 1 Ag^−1^ in 2 M KOH for Co-MoS_2_ nanosheets using a three configuration system was reported [45]. Likewise, two system configurations were used to study the capacitive behavior of Co-MoS_2_ nanoflowers with a specific capacitance of 86 Fg^−1^ at 1 Ag^−1^ in 1 M KOH electrolytes [46]. However, several types of binders were included as part of the active materials in all the aforementioned successful reports of metal-doped MoS_2_-based supercapacitors. The binder is an inactive element that restricts capacitance by contributing its weight to the total mass of the active material. As a result, there is a need to leverage various synthesis approaches that lead to the cost-effective production of binder-free electrodes.

In this work, we report binder-free mixed-phase manganese doped MoS_2_ (Mn-doped MoS_2_) electrodes for an electrochemical performance behavior. The synthesis and characterizations are reported in detail in the subsequent sections, and electrochemical measurements were employed to investigate the capacitive behavior of the electrode materials. The specific capacitance was 70.37 Fg^−1^ at 1 Ag^−1^ in 1 M KOH using three configuration electrode systems. This suggests it as a potential binder-free electrode material for metal-doped MoS_2_-based supercapacitors.

## 2. Materials and Methods

### 2.1. Materials

Manganese (II) acetate tetrahydrate ((CH_3_COO)_2_Mn.4H_2_O, 99%), thiourea (CH_4_N_2_S, 99%), Ammonium molybdate tetrahydrate ((NH_4_)_6_Mo_7_O_24_.4H_2_O, 99%), Nickel foam, mesoporous black carbon, ethanol, Hydrochloric acid (HCl), Potassium hydroxide (KOH), and N-methyl-2-pyrrolidone (NMP) precursors were used. All the chemicals were procured from Sigma Aldrich, Pretoria, Republic of South Africa and used without any further purifications.

### 2.2. Synthesis of Manganese Doped MoS_2_ (Mn-MoS_2_) Nanoflowers

Manganese doped MoS_2_ was synthesized using facile hydrothermally assisted techniques. First, 2.5 g of ammonium molybdate and 4.5 g of thiourea were dissolved in 60 mL of deionized water with vigorous stirring at room temperature. After 30 min of continuous stirring, 0.7 g of manganese (II) acetate tetrahydrate was added to make a composite solution. The resulting solution was aggressively agitated until it became homogeneous. The solution was transferred to a 100 mL Teflon-lined Autoclave and heated at 220 °C for 18 h [47]. After cooling to ambient temperature, the black precipitate was collected by centrifugation. To eliminate any remaining impurities, washing was repeated many times with deionized water and ethanol, then it was dried in a vacuum oven at 80 °C for 12 h. The obtained sample of manganese doped MoS_2_ was denoted MMS.

### 2.3. Preparation of Working Electrodes

A commercially available 1 cm × 3 cm nickel foam was cleaned using 2.0 M HCl, deionized water, and ethanol in a sonication procedure for 15 min before being vacuum dried. To make a slurry, an active substance of Mn-doped MoS_2_ (MMS) was mixed with carbon black (in the ratio 80:20), and appropriate NMP drops and sonicated for 10 min. The slurry was drop cast over cleaned nickel foam and dried for 12 h in a vacuum oven at 80 °C. After drying, the active material on the nickel foam was weighed as approximately 2 mg.

### 2.4. Characterizations

A Rigaku Smartlab X-ray diffractometer (0.154 nm Cu Kα line), field emission scanning electron microscopy (FE-SEM JSM-7800 F, JOEL Ltd, Letchworth, UK), and HORIBA scientific XploRA with LASER light excitation energy (2.411 eV) at 532 nm, were employed to study the crystal structures, morphological and Raman spectroscopic properties of the electrode material, respectively. A surface area prosimetra (TriStar II 3020 version 2.00) was used to determine the Brunauer–Emmett–Teller (BET) surface area.

### 2.5. Electrochemical Measurements

A three-electrode configuration system Autolab PGSTAT302N electrochemical working station was employed to study the electrochemical performance of Mn-doped MoS_2_ in 1 M KOH electrolyte. The three-electrode cells include a reference electrode (Ag/AgCl, 3 M KCl), a counter electrode (platinum wire), and the working electrode (nickel foam). The cyclic voltammetry (CV) curves were measured in a potential window between 0 and 5 V at different scan rates, of 5, 10, 20, 50, and 100 mVs^−1^. The galvanostatic charge–discharge (GCD) measurements were recorded at different current densities of 1, 2, 3, 5, and 10 Ag^−1^. The electrochemical impedance spectroscopy (EIS) was also tested at an amplitude of 5 mV, in the frequency range of 100 kHz to 10 MHz.

## 3. Results and Discussion

**Figure 1:**
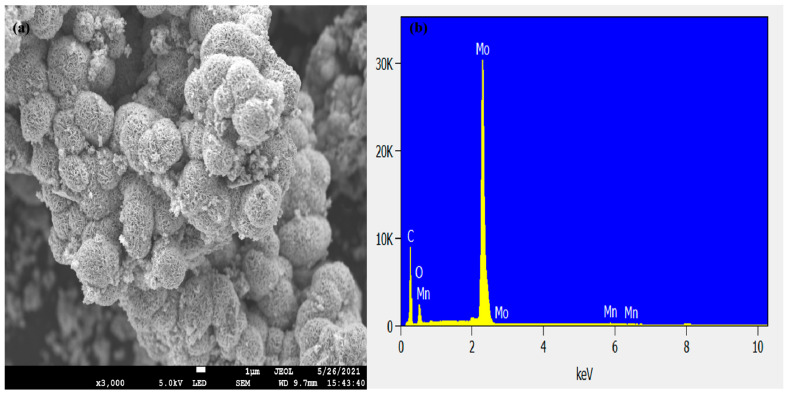
(**a**,**b**): SEM and EDS spectra of the Mn-doped MoS_2_ electrode material.

**Figure 2:**
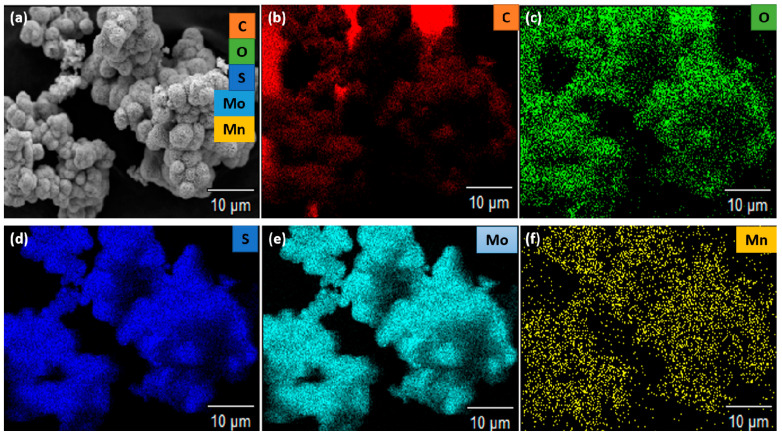
(**a**–**f**): Elemental mapping of the Mn-doped MoS_2_ electrode material.

The morphology and elemental composition of the electrode material were studied using field emission scanning electron microscopy with an EDS analysis system attachment (FE-SEM JSM-7800 F, JOEL Ltd, Letchworth, UK). The as-synthesized materials showed a hierarchical 3D-structure flower-like morphology. The flower-like morphology of the material was highly porous, as shown in Figure 1a. The porosity of the material might be due to the weak Van der Waals force of attraction between the flexible MoS_2_ layers [48,49]. The porosity of the material contributed highly to the performance of the electrochemical behavior. Figure 1b, shows the elemental composition of the prepared sample, which shows the presence of the incorporated Mn. The carbon and oxygen originated from carbon capping of the sample, and the oxygen peak can be traced to the oxygen from the method of preparation, which included water. The molybdenum and sulfur peaks were overlapping, as both elements were operating at the same energy level of 2.29 and 2.30 KeV, respectively. Meanwhile, the appearance of the sulfur element in the mapping images shows its presence in the sample, but this was overlapped on the EDS peaks, due to the proximity of the molybdenum and sulfur energy levels. The elemental distribution of the sample was measured using EDS elemental mapping analysis, recorded at a high magnification of 10 µm. The studies also showed a uniform distribution of the Mo, S, and incorporated Mn, as shown in the mapping analysis of the sample in Figure 2a–f. Table 1 shows the fundamental composition of the sample.

**Figure 3:**
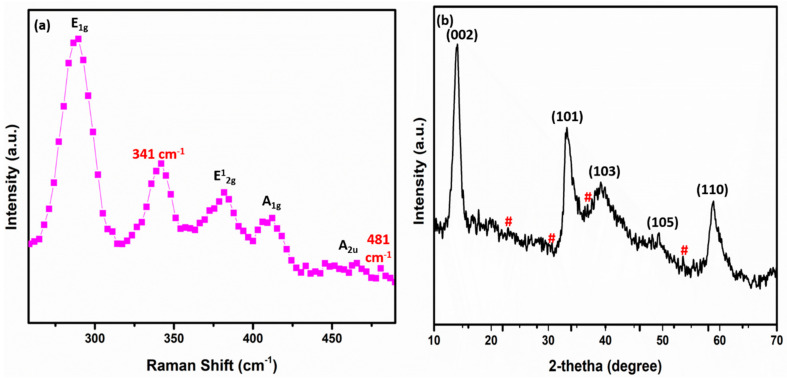
(**a**,**b**): Raman and XRD patterns of the Mn-doped MoS_2_ electrode material. The (#) corresponds to the Mn_2_O_3_ planes of the XRD patterns.

The crystal structure of the sample was further investigated by Raman spectroscopy and X-ray diffraction measurements. Figure 3a shows the pattern of the Raman measurement of the Mn-doped MoS2. The characteristic peaks at 289, 381, 411, and 454 cm^−1^ correspond to the in-plan and out-plane vibrations of E1g, E12g, A1g, and A2u modes of 2H-phase MoS2, respectively. The extra peak of 1T-phase MoS2 was located at 341 cm^−1^, indicating that the as-prepared material was of mixed phases [50]. In addition, an additional peak emerged at 481 cm^−1^, which may belong to local vibrational modes of the Mn2+ dopants [51]. This strongly suggests the successful preparation of the Mn-doped MoS2 nanoflower for electrode material usage. Moreover, Figure 3b depicts the patterns from the XRD measurement results. The corresponding planes (002), (101), (103), (105), and (110) of the 2H-phase MoS2 were associated with the diffraction peaks at about 14.11, 33.31, 39.25, 49.52, and 58.98 degrees (JCPDS No. 37-14920) [49,52]. The high-intensity peak of the (002) plane indicates a well-stacked layer structure along the c-axis. The diffraction peaks represented by the (#) located at angle 23.01, 30.68, 36.83, and 53.58 degrees correspond to the Mn2O3 planes of the (211), (222), (400), and (440) orientations (JSPDS No. 41-1442) [53].

**Figure 4:**
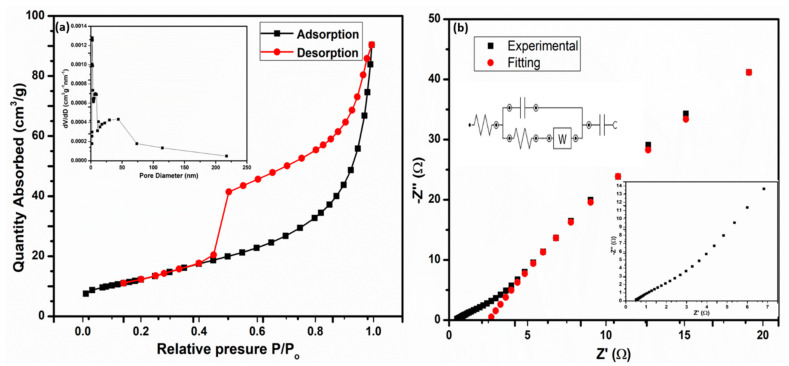
(**a**) N_2_ adsorption–desorption isotherms of Mn-doped MoS_2_, with an inset of its pore size distributions, and (**b**) Nyquist plots (inset lower frequency and equivalent circuit).

Figure 4a shows the N_2_ adsorption–desorption isotherms of Mn-doped MoS_2_, with an inset of its pore size distributions. The electrochemical performance of a supercapacitor was greatly influenced by the surface area of the electrode material. Physical gas adsorption is the preferred approach for evaluating the porous characteristics of electrode materials. The isotherm obtained from these adsorption data was used to calculate the surface area, pore volume, and pore size distribution [54]. The calculated BET results from N_2_ adsorption–desorption isotherms of Mn-doped MoS_2_ have a surface area of 46.0628 m^2^/g. The BET surface area calculated in this study was higher than the previously reported values for bare MoS_2_ (23.9 m^2^/g) [55], which can translate into an improvement in its supercapacitor performance. The linear increase in adsorption at low pressure (P/Po = 0.00–0.10) can be used to identify monolayer gas adsorption inside the pores. After that, up to P/Po = 0.8, the curve reveals a near plateau zone, showing the presence of some nanopores, alongside mesopores. The adsorption of the gas between the interlayers of the sample was indicated by a rapid and spiked increase in the adsorption (P/Po = 0.8–1.0) [56]. As shown in the inset Figure 4a, the pore size distributions of the electrode material were calculated using Barret–Joyner–Halenda (BJH) from adsorption isotherms. The adsorption average pore width (4V/A by BET) for the electrode sample was 11.26607 nm. The cumulative pore volume (BHJ adsorption and desorption) between 1.7000 nm and 300.0000 nm widths for the sample were found to be 0.141921 cm³/g and 0.140928 cm³/g, respectively. Type IV isotherms with a characteristic hysteresis loop indicated a mesoporous structure in the electrode material.

The electrochemical performance of the Mn-doped MoS_2_ electrode material was evaluated in 1 M KOH electrolyte using a three-electrode cell configuration, to conduct CV, GCD, and EIS tests. Figure 4b depicts the related Nyquist plots with their inset lower frequency plot and the equivalent circuits. Electrochemical impedance spectroscopy (EIS) was used to evaluate the electrodes’ improved mechanism. EIS research aids in the development of a theoretical circuit model, for a better understanding of the electrode–electrolyte interface, conductivity calculations, and even electrolyte resistance [45,46]. Over a frequency range of 100 kHz to 10 MHz, with an amplitude of 5 mV and a bias voltage of 0.23 V, EIS was used to measure the ion transport and electrical conductivity of the electrodes. The x-intercept at the high-frequency area of the Nyquist plot gives an equivalent series resistance (ESR), which includes three distinct resistances: (i) resistance of the electroactive material, (ii) resistance of the electrolyte, and (iii) contact resistance at the interface, which was estimated to be around 5.3 Ω. The charge transfer resistance (R_ct_) resulting from the faradic redox process can be calculated using the diameter of the semicircle at a medium frequency. The third region is the Warburg region, which is linked to the redox species diffusion in the sample and mimics the slope of the curve in the low-frequency zone.

**Figure 5:**
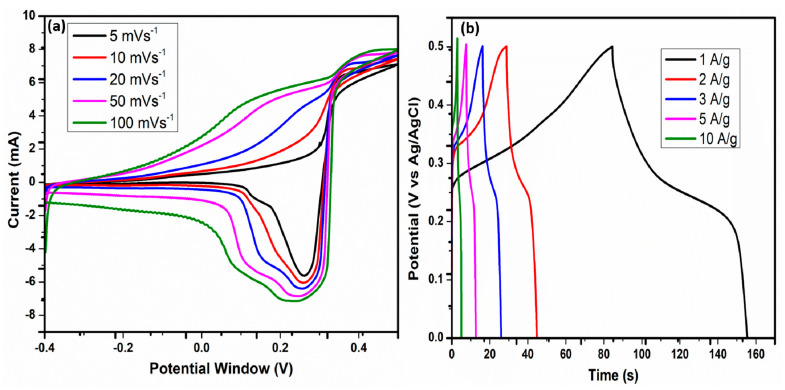
(**a**,**b**): CV and GCD curves of the Mn-doped MoS_2_ Electrode Material.

The CV tests of the Mn-doped MoS_2_ electrode were studied within the potential window of −0.4 V to 0.5 V, with a scan rate between 5 mVs^−1^ to 100 mVs^−1^ (5, 10, 20, 50, and 100 mVs^−1^). As shown in Figure 5a, the CV loops show a typical quasi-rectangular shape with the presence of a redox peak between 0.2 V and 0.4 V in the forward and reverse scans, which may have been due to the different state valences (Mo^4+^/Mo ^(4−∆)+^) from the molybdenum ions. This indicates the ideal pseudocapacitive behavior of the Mn-doped MoS_2_ electrodes, with low contact resistance. The CV loops increased as the scan rate increased, with their curves remaining constant at the highest scan rate of 100 mVs^−1^. The current drawn increased with the voltage as the scan rate increased, increasing the loop area. The electrodes’ fast response and charge–discharge capabilities were also noticed, as well as a clear distinction between the cathodic (reduction) peaks of the electrodes’ CV loops. The pseudo-capacitance behavior was confirmed by the presence of redox peaks in the composite. The pseudocapacitive performance was due to the Faradaic reactions that occurred at the electrode–electrolyte interface [57].

GCD plots of the Mn-doped MoS_2_ electrode material are shown in Figure 5b. The GCD studies of the electrode material were conducted at different current densities of 1, 2, 3, 5, and 10 Ag^−1^. The GCD curves have nearly perfect triangular forms, with a small reduction in internal resistance at the onset of discharge curves, which could have been due to the active materials’ contact resistance with the highly conductive nickel foam surface. In addition, as seen in semi-symmetry curves of the GCD, the oxidation and reduction processes at the electrode–electrolyte border of the electrode materials indicated the pseudo-capacitance behavior of the electrodes [58]. The specific capacitances were calculated from the GCD curves, using $C_{sp}=\frac{I\Delta t}{m\Delta V}$ to determine the performance parameters of the supercapacitor electrode. The Mn-doped MoS_2_ electrode showed the specific capacitances of 70.37, 58.89, 57.69, 44.11, and 29.90 Fg^−1^ at the current densities of 1, 2, 3, 5, and 10 Ag^−1^, respectively. The highest observed specific capacitance of the electrode was 70.37 Fg^−1^ at a 1 Ag^−1^ current density.

The highest specific energy and power densities obtained were calculated using both E=12CsΔV23.6 and $P=\frac{3600\;\times \;E}{\Delta t}$ equations, as 3.14 Wh/kg and 4346.35 W/kg, respectively. At the current density of 1 Ag^−1^, a longer period of discharge was observed, which may have been due to the electrode’s surface area. Furthermore, when the current densities increased, the discharge time decreased, which corresponds to the stated CV data. The charge storage in the material occurred because of either a surface-limited non-faradaic reaction (i.e., adsorption) or the intercalation of electrolyte ions into the material’s interlayer spacing [59]. The performance parameters of the galvanostatic charge–discharge curve are presented in Table 2. As shown in Table 2, the energy density decreased as the current density increased, due to their proportionality to the specific capacitance. In addition, while the contact between the electrolyte and electrode was limited, the power density increased with the scan rate, because it offered less resistance at higher scan rates.

**Figure 6:**
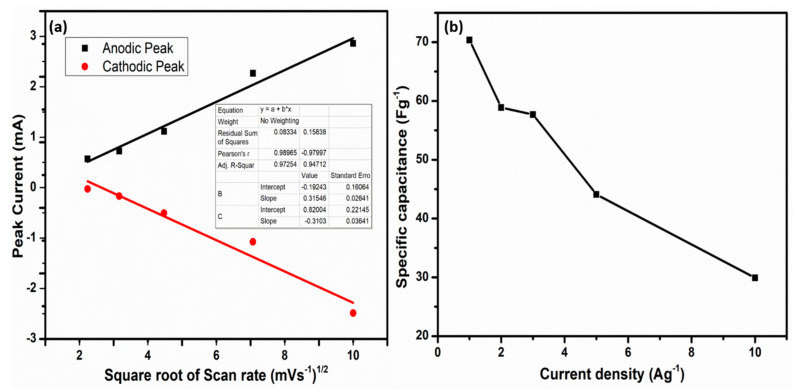
(**a**) Peak current against the square root of scan rate, and (**b**) specific capacitance comparison with current density.

The anodic and cathodic peak current measurement, as shown in Figure 6a, was directly proportional to the square root of the scan rate, further corroborating the electrode materials’ pseudocapacitive behavior as an appropriate composite for supercapacitor applications [60]. The intercept, slope, and R. square values of both the anodic and cathodic peaks are shown in the inset of Figure 6a. The capacitances dramatically decreased as the current density increased, as shown in Figure 6b, indicating an exceptional ability for fast charge–discharge; and the restriction of ions on the electrode materials’ surface could be responsible.

## 4. Conclusions

A mixed-phase Mn-doped MoS_2_ nanoflower was synthesized using a one-pot facile hydrothermal technique. The elucidation of the as-synthesized material was carried out using XRD, RAMAN, SEM-EDS, and BET methods. The presence of a mixed-phase in the as-synthesized electrode material was established from the XRD and RAMAN spectrum results. The nanoflower morphology of the Mn-doped MoS_2_ was also established from the SEM-EDS images, and the surface of areas with the presence of a mesoporous structure were likewise revealed from the BET techniques. The supercapacitor performance of the fabricated supercapacitor electrode was electrochemically investigated using cyclic voltammetry, galvanostatic charge–discharge, and electrochemical impedance spectroscopy measurements in a three-electrode cell system. The Mn-doped MoS_2_ nanoflower exhibited a specific capacitance of 70.37 Fg^−1^ at a current density of 1 Ag^−1^, with corresponding energy and power densities of 3.14 Whkg^−1^ and 4346.35 Wkg^−1^, respectively. The electrode’s performance shows that a mixed-phase Mn-doped MoS_2_ could be an outstanding material for metal-doped MoS_2_ supercapacitive electrodes. Smart device fabrication and further optimization of the electrode performance, such as the characterization of the electrodes before and after cycles, are recommended for future work, for a better understanding of the electrode–electrolyte interface of the materials.

## Figures and Tables

**Table 1:** Quantitative composition of the elements presents in the Mn-doped MoS_2_.

Element Line	Weight %	Norm. Wt.%	Atom %	Formula	Compnd %	Norm. Compnd. %
*C K*	26.7	26.7	57.3	C	26.7	26.7
*O K*	16.5	16.5	26.4	O	16.5	16.5
*Mn K*	5.4	5.4	2.5	Mn	5.4	5.4
*Mo L*	51.4	51.4	13.8	Mo	51.4	51.4
*Total*	100.0	100.0	100.0		100.0	100.0

**Table 2:** Performance Parameters of the Galvanostatic Charge–Discharge.

Current Density (A/g)	Specific Capacitance (F/g)	Energy Density (Wh/kg)	Power Density(W/kg)
1	70.37	2.07	257.14
2	58.89	2.29	529.69
3	57.69	2.31	805.85
5	44.11	2.58	1454.52
10	29.90	3.14	4346.35

## Data Availability

The data presented in this study are available on request from the corresponding author.
